# Establishment of a Three-Dimensional In Vitro Model of Taste Bud Cells for Fat Taste Investigation

**DOI:** 10.3390/ijms27198545

**Published:** 2026-09-24

**Authors:** Sidy Saidou-Diabé, Amira Sayed Khan, Naim Akhtar Khan, Aziz Hichami

**Affiliations:** Physiologie de la Nutrition & Toxicologie, INSERM, CTM UMR U1231, Université Bourgogne Europe, 6 Boulevard Gabriel, 21000 Dijon, France

**Keywords:** fat taste, taste buds, CD36, 3D culture, spheroids

## Abstract

Fat taste is emerging as a sixth primary taste modality, with CD36 identified as a key receptor for oral detection of long-chain fatty acids. Tastants bind to receptors on taste bud cells (TBCs), triggering intracellular calcium mobilization, neurotransmitter release, activation of gustatory afferent fibers, and transmission of sensory information to the brain. Conventional two-dimensional (2D) culture systems fail to reproduce the complex three-dimensional (3D) architecture and cell-to-cell interactions essential in vivo. Here, we developed a 3D model of mouse TBCs to investigate the cellular mechanisms underlying fat taste perception. Immortalized CD36-positive TBCs and circumvallate papilla-derived TBCs were co-cultured under 2D and 3D conditions. In 3D conditions, TBCs self-organized into spheroids displaying organelles, intercellular junctions, and expression of markers of the three major TBC types. Compared to 2D-cultured cells, spheroids exhibited higher expression of taste receptors, including CD36, sweet receptor T1R2, umami receptor T1R1. Wnt/β-catenin and Sonic Hedgehog pathways, controlling differentiation, were upregulated. Spheroids showed enhanced responses to linoleic acid, with greater intracellular calcium mobilization and serotonin release. This model recapitulates more faithfully structural and functional properties of native taste tissue, providing a physiologically relevant in vitro platform for studies of fat taste mechanisms and potential factors modulating TBC function.

## 1. Introduction

Taste is a critical sensory modality for survival, as it promotes the consumption of energy-dense nutrients while enabling the avoidance of potentially harmful substances. To date, five primary taste modalities have been established: sweet, salty, bitter, sour, and umami. Over the past two decades, fat taste or “oleogustus” has emerged as a putative sixth basic taste modality [1,2]. Lingual taste papillae constitute the principal sites of taste perception and are classified into three types in mammals: fungiform papillae located anteriorly, foliate papillae on the lateral edges, and circumvallate papillae at the posterior region of the tongue. Each taste papilla contains multiple taste buds, which are composed of approximately 50–100 taste bud cells (TBCs). Taste buds comprise four main cell types: type I cells, which are glial-like and primarily involved in support functions and salt detection; type II cells, or receptor cells, which detect sweet, bitter, umami, and fat tastants; type III cells, or presynaptic cells, which form conventional synapses with gustatory neurons and release neurotransmitters such as noradrenaline and serotonin; and type IV cells, which act as progenitors for the other three cell types [3]. Type I, II, and III cells extend toward the gustatory pore at the surface of the tongue, where they are exposed to tastants. Type I cells ensheathe type II and III cells, while type IV cells are located at the base of the taste bud.

Several taste receptors involved in the detection of long-chain fatty acids (LCFAs), including GPR40 (also known as FFAR1), GPR120 (FFAR4), and Cluster of Differentiation CD36 (CD36), have been described [4,5]. Lingual expression of GPR40 has only been reported in mice [4]. Both humans and rodents exhibit a spontaneous preference for fatty foods, which involves at least two identified lingual lipid sensors, namely CD36 and GPR120. Accumulating evidence indicates that CD36 is the primary fat taste receptor involved in the detection of LCFAs, whereas GPR120 appears to play a distinct role in the post-oral regulation of food intake and exhibits a lower affinity for LCFAs compared to CD36 [2].

Altered expression of lingual CD36 protein modulates feeding behaviour and, consequently, contributes to the development of obesity. In rodent models, exposure to a high-fat diet leads to downregulation of CD36 expression in taste buds, which is accompanied by reduced sensitivity to dietary lipids [6]. Consistently, in humans, obesity is associated with decreased CD36 expression, correlating with impaired orosensory fat detection [2]. At the genetic level, several studies across diverse populations have identified *CD36* polymorphisms, particularly rs1761667, as being associated with obesity and diminished fat taste perception [7,8]. Moreover, epigenetic modifications of *CD36*, including CpG methylation, have been positively correlated with obesity and reduced lipid taste sensitivity [9,10]. Cellular in vitro studies showed that human taste bud cells transfected with CD36 siRNA had a lower response to linoleic acid than human taste bud cells transfected with nontargeting siRNA and the same results were found in taste bud cells from CD36 KO mice [11].

In mice, CD36 is predominantly expressed in the circumvallate papillae (CVP) [12]. However, given that mice possess only a single CVP, experimental investigations of fat taste require the use of a large number of animals. This constitutes a significant ethical limitation and underscores the need for the development of alternative experimental models to study fat taste.

In vitro studies of taste commonly rely on two-dimensional (2D) culture systems, which fail to reproduce the complex intercellular communication observed in native tissues composed of multiple interacting cell types [13].

For example, within taste buds, type II cells respond to tastants (sweet, bitter, umami) by increasing intracellular Ca^2+^ and releasing ATP. This ATP not only activates afferent nerve fibers but also stimulates adjacent type III (presynaptic) cells, illustrating a key paracrine signaling pathway. ATP is rapidly degraded by NTPDase2 expressed on type I cells, thereby tightly regulating its extracellular availability [14]. In turn, type III cells release serotonin (5-HT), either in response to ATP or direct stimulation, establishing a feedback loop within the taste bud. Specifically, 5-HT acts back on type II cells via 5-HT_1_A receptors to exert negative feedback, while ATP can also act in an autocrine manner through purinergic receptors to sustain excitation [15].

In the context of fat taste, we showed that polyunsaturated fatty acids (PUFAs), such as linoleic acid, induce an increase in intracellular Ca^2+^ in CD36^+^ taste bud cells, leading to the release of serotonin [16]. These observations further support the idea that lipid sensing engages Ca^2+^-dependent signaling pathways and contributes to intercellular communication within taste buds.

Together, these interactions-mediated by ATP and other neurotransmitters, as well as locally produced peptides and hormones such as GLP-1, neuropeptide Y, and cholecystokinin—highlight a highly coordinated network of autocrine and paracrine signaling within taste buds. Furthermore, the presence of gap junctions between taste bud cells suggests that electrical coupling may represent an additional mechanism of intercellular communication [17]. This complex signaling environment integrates multiple pathways to fine-tune taste perception and cellular responses. Such multidimensional communication is largely absent in 2D culture systems, underscoring their limitations for accurately modelling taste physiology.

Three-dimensional (3D) culture systems have emerged as alternative approaches for modelling tissue organization and cellular interactions [13]. Organoids have also been established in several tissues, demonstrating their potential for modelling tissue development, disease, and cellular differentiation [18,19,20]. Organoid approaches have enabled the generation of multicellular taste structures from Lgr5^+^ and Lgr6^+^ progenitor cells, containing proliferative and differentiated taste cells capable of responding to tastants [21,22,23,24,25,26,27]. However, conventional taste organoids are frequently generated within extracellular matrices such as Matrigel, which may introduce variability and limit direct access of taste receptor cells to external tastants [23,24,25,26,27,28].

Spheroids represent a complementary scaffold-free 3D culture approach in which established cells self-aggregate under non-adherent conditions. Compared with conventional 2D cultures, spheroids can promote enhanced cell–cell interactions and tissue-like organization while offering relative simplicity and reproducibility [29]. Immortalized human taste bud cells have been shown to express CD36 and respond to linoleic acid [30], and human taste spheroids have demonstrated functional calcium responses to bitter compounds [31]. However, to our knowledge, an equivalent murine scaffold-free taste spheroid model specifically designed to investigate fat taste has not yet been established.

While sweet, salty, bitter, umami, and sour tastes have been investigated in 3D taste models responses [18], fat taste remains poorly explored in such systems. Therefore, in the present study, we established a scaffold-free 3D spheroid model based on immortalized CD36^+^ murine taste bud cells and circumvallate papillae-derived cells. We characterized the resulting spheroids at the structural, molecular, and functional levels and compared them with conventional 2D cultures, with particular emphasis on lipid sensing and responses to linoleic acid. This model was developed to provide a relevant in vitro platform for investigating the cellular and molecular mechanisms underlying fat taste while reducing reliance on animal experimentation.

## 2. Results

We established 3D-cultured taste bud cells in organoid-developing medium (conditioned DMEM/F12) from 2 immortalized taste bud cell lines, CD36+ and whole circumvallate papillae-derived cells. 2D-cultured cells with the typical taste bud cells medium (conditioned IMDM) were our control and we compared these two conditions in many aspects, including their overall morphology, gene expression, calcium signaling in response to tastants and serotonin secretion in response to linoleic acid.

### 2.1. 3D-Cultured Cells Form Spheroids with Numerous Intercellular Connexions and Secretion Vesicles

To investigate the structural organization of the spheroids and evaluate the interactions among the different cell populations composing these 3D aggregates, scanning electron microscopy (SEM) and transmission electron microscopy (TEM) analyses were performed.

SEM examination (Figure 1) revealed that the spheroids were compact, spherical structures with an average diameter of approximately 220–250 µm. Higher-magnification images of the outer spheroid layer showed cells of heterogeneous sizes and morphologies, suggesting the presence of multiple cellular subpopulations within the spheroids (Figure 1A). Numerous intercellular connections were observed between adjacent cells (Figure 1A–C), indicating extensive cell–cell interactions and structural cohesion within the 3D architecture. In addition, the cell surface appeared irregular and textured (Figure 1A,B), a characteristic commonly associated with active cellular communication and extracellular matrix deposition.

Further ultrastructural characterization by TEM (Figure 2) revealed several features indicative of high cellular activity. Spheroids contained abundant mitochondria, numerous intracellular and extracellular secretory vesicles (Figure 2A–D), and a well-developed endoplasmic reticulum (Figure 2C), suggesting elevated metabolic and protein synthesis activities. The secretory vesicles observed by TEM had a mean diameter of approximately 70 nm, ranging from 55.8 to 81.7 nm (Supplementary Figures S1 and S2). Furthermore, the presence of desmosomal junctions (Figure 2C) confirmed the establishment of strong intercellular adhesion complexes, contributing to spheroid integrity and tissue-like organization. Collectively, these ultrastructural observations demonstrate that the spheroids constitute a highly organized multicellular system displaying active metabolic, secretory, and intercellular communication processes.

### 2.2. 3D-Cultured Cells Express the 3 Main Types of TBCs

Haematoxylin and eosin (H&E) staining revealed a compact spheroidal structure with a relatively homogeneous cellular distribution and limited intercellular spaces (Figure 3A).

To assess the presence of the major taste bud cell (TBC) populations within the spheroids, immunofluorescence staining was performed using antibodies against glutamate-aspartate transporter (GLAST), a marker of type I cells, α-gustducin, a marker of type II cells, synaptosome-associated protein 25 (SNAP-25), a marker of type III cells, and CD36, a fatty acid receptor. Nuclei were counterstained with DAPI.

GLAST immunoreactivity was detected throughout the spheroids, including both peripheral and central regions (Figure 3F). In contrast, α-gustducin-positive cells (Figure 3B) and SNAP-25-positive cells (Figure 3F) were mainly observed in the outer layers of the spheroids. CD36 staining was detected in both the peripheral and inner regions and showed partial co-localization with α-gustducin-positive cells (Figure 3C). These observations are consistent with previous reports describing CD36 expression in type II taste bud cells [12].

Quantitative analysis using QuPath showed that 49.43% of the analyzed cells were GLAST-positive (172/348), 41.63% were α-gustducin-positive (403/968), including 32.64% α-gustducin+/CD36+ cells (316/968), and 5.48% were SNAP-25-positive (43/785), confirming the presence of the three major taste bud cell populations within the 7-day spheroids, in agreement with the relative proportions reported for native taste buds (Figure 3H–J).

### 2.3. 3D-Culture Conditions Enhance mRNA Expression of Taste Receptors, Wnt and Shh Signaling Cascades

To assess the impact of culture conditions on the expression of taste receptor genes as well as genes involved in cell proliferation and differentiation, quantitative RT-PCR analyses were performed on cells cultured under 2D and 3D conditions (Figure 4 and Figure 5).

The expression of several taste receptor genes was increased in 3D-cultured cells compared to 2D-cultured cells (Figure 4). This included *CD36* and *GPR120* (Figure 4A,B), two receptors involved in long-chain fatty acid sensing, as well as Taste receptor type 1 member 1, *T1R1* (Figure 4C), a subunit of the umami taste receptor, Taste receptor type 1 member 2, *T1R2* (Figure 4D), a subunit of the sweet taste receptor, and Taste receptor type 2 member 38, *T2R38* (Figure 4E), a bitter taste receptor. Among the genes analyzed, *CD36* exhibited the highest increase in expression in spheroids, with an approximately 60-fold higher mRNA level than in monolayer cultures. *T1R1* showed the second highest increase, with an approximately 11-fold upregulation. Increased expression of *GPR120*, *T1R2*, and *T2R38* was also observed in 3D-cultured cells compared with 2D-cultured cells.

Proliferation marker *Ki67* was expressed at lower levels in 3D-cultured cells than in 2D-cultured cells, with mRNA levels reduced by approximately 50% in spheroids (Figure 5A).

Expression of genes associated with the Wnt and Sonic Hedgehog (Shh) signaling pathways, which are involved in taste cell proliferation and differentiation [32], was also examined (Figure 5B–E). *β-catenin* and *Lgr5* (Figure 5B,C), two components of the Wnt signaling pathway, were upregulated in 3D cultures compared with 2D cultures, showing approximately 2-fold and 15-fold increases, respectively. Similarly, the expression of *Gli1* and *Sox2* (Figure 5D,E), genes associated with the Shh pathway, was higher in spheroids, with approximately 3-fold and 25-fold increases, respectively.

Overall, cells cultured under 3D conditions displayed higher expression levels of taste receptor genes and genes associated with the Wnt and Shh signaling pathways, whereas *Ki67* expression was lower than in cells cultured under 2D conditions.

### 2.4. 3D-Culture Conditions Enhance Calcium Signaling in TBCs in Response to Different Tastants

To determine whether the increased expression of taste receptor genes observed in spheroids was associated with enhanced functional responses to tastants, intracellular calcium mobilization assays were performed. Since changes in intracellular calcium concentration ([Ca^2+^]i) represent a key event in taste signal transduction, calcium responses were measured in cells stimulated with taste compounds.

Cells cultured under 2D and 3D conditions were dissociated using Accutase, left for 18 h, loaded with the calcium-sensitive fluorescent dye Fluo-4, and stimulated with either linoleic acid, a long-chain fatty acid, or monosodium glutamate (MSG), an umami tastant (Figure 6). Linoleic acid or MSG was added after 2 min of baseline recording. The concentrations of LA and MSG used in the present study were selected based on preliminary concentration-response experiments performed in 2D- and 3D-cultured taste bud cells. Concentrations were selected to produce clear and significant differences between 2D- and 3D-cultured cells while avoiding excessive stimulation. Accordingly, 25 µM LA and 75 µM MSG were selected for calcium imaging. Stimulation with 25 µM linoleic acid induced an increase in intracellular calcium levels in both culture conditions (Figure 6A). However, the maximal fluorescence intensity was higher in cells derived from spheroids than in cells cultured as monolayers, reaching approximately 40% and 25% above baseline, respectively (Figure 6B).

Similarly, stimulation with 75 µM MSG elicited an increase in intracellular calcium levels in both groups (Figure 6C). Cells derived from 3D cultures exhibited a trend towards a greater response, with a fluorescence increase of approximately 20%, whereas cells from 2D cultures showed an increase of approximately 11%.

Quantification of the maximal calcium responses confirmed significantly higher (ΔF/F_0_)_max_ values in cells derived from 3D cultures following stimulation with linoleic acid but not with MSG (Figure 6B,D).

### 2.5. 3D-Culture Conditions Enhance Serotonin Secretion in Response to Linoleic Acid

To further assess the functional properties of the cultured cells, we investigated whether the increase in intracellular calcium concentration ([Ca^2+^]i) induced by tastant stimulation was associated with neurotransmitter release. We therefore analyzed the expression of genes involved in serotonin synthesis and vesicular transport and quantified serotonin secretion following stimulation with linoleic acid.

Aromatic L-amino acid decarboxylase (AADC) catalyzes the conversion of 5-hydroxytryptophan into serotonin, whereas vesicular monoamine transporter 2 (VMAT-2) mediates the transport of monoamines from the cytosol into secretory vesicles. Quantitative RT-PCR revealed higher expression levels of *AADC* and *VMAT-2* in 3D-cultured cells compared with 2D-cultured cells, with respectively a 4-fold and a 2-fold increases (Figure 7A,B).

Serotonin release was quantified by ELISA following a 10 min stimulation with either a control solution or 100 µM linoleic acid. A concentration-response experiment comparing 2D- and 3D-cultured cells (Supplementary Figure S3) showed that 100 µM linoleic acid produced the highest serotonin secretion and the most pronounced difference between the two culture conditions; this concentration was therefore selected for the experiments presented in Figure 7C. Serotonin concentrations were normalized to total cellular protein content (Figure 7C). Under control conditions, serotonin secretion was similar in 2D- and 3D-cultured cells, reaching 0.022 pg/µg and 0.018 pg/µg of protein, respectively. However, following stimulation with 100 µM linoleic acid, cells derived from 3D cultures secreted significantly higher amounts of serotonin than cells cultured in 2D, with values of 0.15 pg/µg and 0.09 pg/µg of protein, respectively, corresponding to an 8-fold and a 4-fold increase compared to basal conditions (Figure 7C). This highlights that 3D culture conditions favour responses to tastants, through basal upregulation of neurotransmitter enzymes and increased neurotransmitter release in response to tastants.

## 3. Discussion

In the present study, we developed a scaffold-free three-dimensional (3D) spheroid model to investigate taste bud cell biology, with a particular focus on fat taste. To enrich the model in cells involved in lipid sensing, spheroids were generated by co-culturing two populations of immortalized murine taste bud cells at a 1:1 ratio: CD36-positive taste bud cells and cells derived from whole circumvallate papillae. The resulting spheroids were subsequently characterized at the structural, molecular, and functional levels. Quantitative Live/Dead analysis confirmed the high viability of the spheroids, with 99.07% (Supplementary Figure S4) and 99.30% (Supplementary Figure S5) of cells remaining viable after 7 and 14 days of culture, respectively, supporting their stability during prolonged culture. Spheroids exhibited a compact multicellular organization, numerous organelles and contained the three major taste bud cell populations. Compared with conventional two-dimensional (2D) cultures, they displayed increased expression of several taste receptor genes, including the lipid receptors *CD36* and *GPR120*, together with enhanced calcium signaling and serotonin release in response to linoleic acid stimulation. CD36 and GPR120 appear to play distinct but complementary roles in fatty acid sensing, with CD36 acting as a primary high-affinity receptor for the initial detection of dietary fatty acids, whereas GPR120 contributes mainly to lipid signaling at higher fatty acid concentrations [2,11]. Based on the established role of CD36 in fatty acid detection, we therefore selected CD36-positive cells as the cellular basis for developing our 3D spheroid model of lipid sensing. However, the respective contributions of CD36 and GPR120 to the linoleic acid response cannot be quantitatively determined from mRNA expression levels alone and would require receptor-specific functional experiments. Overall, these findings indicate that the spheroids provide a differentiated and functionally responsive model enriched in lipid-sensing taste cells.

The transition from 2D to 3D culture systems has markedly improved the physiological relevance of in vitro models by promoting cell–cell interactions and tissue-like organization [13]. Consistent with observations in other cell types, immortalized taste bud cells cultured under non-adherent conditions spontaneously self-organized into compact spheroids. Our spheroids were approximately 220–250 µm in diameter. At this size, spheroids are generally considered to remain below the threshold of 500 µm, at which hypoxic or necrotic cores develop [33,34], which is consistent with our histological observations. Ultrastructural analyses further revealed abundant mitochondria, endoplasmic reticulum, secretory vesicles, and numerous cell–cell contacts, indicating that cells remained viable and maintained a high degree of structural organization within the spheroids. Similar ultrastructural features have been reported in native taste buds: in particular, Inoko and colleagues described rat taste bud cells containing numerous intracellular vesicles and exhibiting specialized intercellular contacts, including synaptic structures and subsurface cisternae associated with taste signal transmission [35]. Likewise, our spheroids displayed abundant intracellular and extracellular vesicles together with numerous cell–cell contacts, suggesting that they reproduce several ultrastructural characteristics of native taste tissue.

Immunofluorescence analyses demonstrated the presence of the three major taste bud cell populations within the spheroids, including GLAST-positive type I cells, α-gustducin-positive type II cells, and SNAP-25-positive type III cells. Interestingly, type II and type III cells were predominantly localized at the periphery of the spheroids, whereas type I cells were distributed throughout both the peripheral and central regions. Similar spatial heterogeneity has been reported in other multicellular spheroid models and is generally attributed to gradients of nutrients, oxygen, and local signaling molecules that arise during three-dimensional growth, resulting in distinct cellular microenvironments between the spheroid core and periphery [33]. In the present model, such organization may favor the localization of sensory receptor cells, types II and III, at the outer layer, while support cells, type I, remain distributed throughout the structure, although the mechanisms underlying this spatial arrangement remain to be elucidated.

The absence of an extracellular matrix scaffold may also contribute to this organization. Most previously described taste bud organoid models rely on extracellular matrices such as Matrigel to support cell growth and differentiation [24,25,26,27]. Although Matrigel provides a favorable microenvironment, its poorly defined composition, batch-to-batch variability, and animal origin may limit reproducibility [36]. In conventional taste bud organoids generated within Matrigel, the apical pole with taste receptor cells is located on the inner layers of the organoid, making them largely inaccessible to tastants and requiring dissociation before functional assays. This is the case for taste bud organoids described in mice [24,25,26,27,28], pigs [37], and non-human primates [38]. Consequently, several groups have developed polarity-reversal strategies based on the removal of Matrigel and subsequent suspension culture to generate “apical-out” organoids, thereby exposing taste receptor cells to the external environment and enabling direct functional analyses [28,39]. Adpaikar and colleagues [28] showed that suspension culture of Matrigel-derived taste organoids induces apicobasal polarity reversal, resulting in the outward localization of the apical pole and improved accessibility for calcium imaging experiments. Beyond the study of fat taste, previous scaffold-free 3D taste models have also been used to investigate other taste modalities, including sweet, bitter, sour, salty, and umami responses [18]. These studies demonstrated that suspension-cultured taste organoids can retain functional taste responses and provide a suitable platform for investigating taste-related cellular signaling in a three-dimensional context.

The present study shows that immortalized murine taste bud cells can directly self-assemble into scaffold-free spheroids under non-adherent conditions, without the need for Matrigel embedding or subsequent polarity-reversal procedures. Importantly, taste receptor cells (type II, α-gustducin-positive) and presynaptic cells (type III, SNAP-25-positive) were localized at the spheroid periphery, making them readily accessible to stimulation and functional analyses. Beyond simplifying culture procedures, this scaffold-free configuration may therefore provide a useful platform for investigating taste transduction mechanisms.

One of the most striking findings of this study was the marked increase in taste receptor gene expression observed in spheroids. Expression of *CD36*, *GPR120*, *T1R1*, *T1R2*, and *T2R38* was significantly higher in 3D-cultured cells than in monolayer cultures, with CD36 showing the strongest upregulation. These findings suggest that 3D culture conditions promote the maintenance or acquisition of a more differentiated taste-cell phenotype compared with conventional 2D cultures. Similar observations have been reported in other cellular models, where three-dimensional organization favors cellular specialization and tissue-specific functions. For example, periodontal ligament stem cells cultured as spheroids displayed enhanced osteogenic differentiation compared with cells maintained in monolayer cultures [40], likewise 3D-organized cardiac cells exhibited reduced proliferation, increased expression of differentiation markers, and greater functional maturation than their 2D counterparts [41]. These observations are consistent with our findings showing increased expression of taste receptor genes together with reduced *Ki67* expression in spheroids.

In parallel with the reduction in proliferation, spheroids also exhibited increased expression of genes associated with the Wnt and Sonic Hedgehog (Shh) signaling pathways. Both pathways are known to play essential roles in taste bud renewal, progenitor-cell proliferation, and taste-cell differentiation [42]. In particular, Lgr5-positive progenitor cells constitute a major source of taste-cell generation in vivo and in organoid cultures [22,23]. Consistent with these observations, spheroids showed increased expression of *β-catenin* and *Lgr5*, parts of Wnt signaling pathway, *Gli1* and *Sox2*, parts of Shh signaling pathway suggesting activation of molecular programs involved in stem-cell maintenance and taste-cell differentiation. Although the causal relationship between activation of these signaling pathways and the molecular changes observed in spheroids remains to be established, the three-dimensional microenvironment itself may contribute to these effects. Increased cell–cell interactions and cellular density are known to influence stem-cell behavior and differentiation through the modulation of signaling pathways such as Wnt. Such findings have been reported in other stem-cell models, including rat mesenchymal stem cells and human neural stem/progenitor cells, in which cell aggregation or high-density culture promoted differentiation and altered Wnt-related signaling [43,44]. These observations support the hypothesis that the enhanced cell–cell communication and tissue-like organization achieved in spheroids favor the activation of signaling networks associated with differentiation and functional maturation.

A major advantage of the spheroid model is its ability to maintain cells in culture for longer periods than conventional monolayer cultures while preserving a stable three-dimensional architecture. While 2D cultures reached confluence after approximately 3 days and detached from the plates after, spheroids remained viable and structurally organized for at least 14 days, thereby providing an extended period for cellular maturation. Prolonged culture has been shown to promote functional maturation in several in vitro models [45,46,47,48]. In addition to the three-dimensional architecture, spheroids were maintained in a serum-free conditioned DMEM/F12 medium, which is routinely used for the generation and maintenance of taste bud organoids [24,25,26,27,28]. This defined medium contains growth factors that regulate taste stem cell maintenance and differentiation, including R-spondin 1, a key activator of the Wnt signaling pathway [49], and Noggin, a BMP antagonist that promotes taste cell differentiation [32]. Together, the prolonged culture period, the tissue-like 3D microenvironment that enhances cell–cell interactions, and the presence of differentiation-promoting growth factors likely contributed to the more differentiated phenotype observed in spheroids. An additional consideration is that the differences observed between 2D and 3D cultures may not be attributable exclusively to three-dimensional organization, as the two culture systems also differed in medium composition and culture duration. To further assess the potential contribution of the culture medium, we performed additional matched-medium experiments in which 2D and 3D cultures were maintained either in completed IMDM or completed DMEM/F12 (Supplementary Figure S6). Under both medium conditions, the overall molecular profile remained more pronounced in 3D cultures, with several taste-related and differentiation-associated markers showing higher expression in 3D than in 2D cultures. These observations support the interpretation that the differences between the two culture systems cannot be explained solely by medium composition, although the magnitude of some molecular changes may be influenced by the culture medium. However, because 2D cultures were maintained for 3 days whereas spheroids were analyzed after 7 days, the respective contributions of three-dimensional organization, culture duration, and medium composition cannot be completely separated in the present study. The matched-medium experiments are therefore provided as Supplementary Figure S6, while a more comprehensive experimental design will be required to independently assess the contribution of each factor.

Beyond the molecular changes observed in spheroids, the 3D culture system also displayed enhanced functional responses to tastants. To compare individual cell responses, spheroids and 2D-cultured cells were dissociated with Accutase before functional analyses. Following dissociation, cells were replated and allowed to recover for 18 h before calcium imaging experiments to ensure stable adhesion and limit potential artefacts related to the dissociation procedure. Under these conditions, cells derived from 3D cultures exhibited larger intracellular calcium responses to linoleic acid significantly and monosodium glutamate than cells maintained in conventional 2D cultures. These functional observations are consistent with the increased expression of *CD36*, *GPR120*, and *T1R1* observed at the transcript level. In contrast, no significant difference was detected in the calcium response to saccharose despite the increased expression of T1R2, which forms the sweet taste receptor heterodimer with T1R3. This apparent discrepancy may reflect heterogeneity in receptor expression among cells within the spheroids or indicate that increased *T1R2* transcript abundance alone is insufficient to enhance receptor function.

Because intracellular Ca^2+^ mobilization is a pivotal step in taste signal transduction, we next examined whether the enhanced calcium responses were associated with neurotransmitter release. Type II taste bud cells respond to tastants by increasing intracellular Ca^2+^ and releasing ATP, which can activate gustatory afferent nerve fibers and stimulate neighboring type III presynaptic cells. These type III cells, in turn, release serotonin, thereby contributing to gustatory signal transmission and modulation. In line with this mechanism, we found that *AADC* and *VMAT-2*, two genes involved in serotonin synthesis and vesicular transport, were upregulated in spheroids. Furthermore, linoleic acid elicited significantly greater serotonin release in 3D-cultured cells than in 2D-cultured cells, while basal serotonin secretion remained unchanged. These findings are consistent with previous reports showing that native CD36-positive taste bud cells release serotonin in response to long-chain fatty acids [16]. CD36-positive taste bud cells are also reported to express type II markers such as α-gustducin and PLCβ2, suggesting an association with the type II receptor-cell lineage [12]. However, their capacity to secrete neurotransmitters may indicate atypical functional properties, and it remains possible that CD36-positive cells represent a heterogeneous population with overlapping type II- and type III-like features. These results demonstrate functional maturation of type III–like cells but do not resolve the relative contribution of paracrine ATP signaling versus intrinsic cellular changes. Furthermore, extracellular serotonin levels reflect the balance between serotonin synthesis and release, as well as its degradation, potentially through monoamine oxidase (MAO) activity; however, MAO-dependent degradation was not directly assessed in the present study.

Although the present model recapitulates several key features of taste bud biology, it remains a simplified representation of the native gustatory environment. In vivo, taste bud cells reside within a complex microenvironment where they continuously interact with gustatory neurons, von Ebner gland secretions, immune cells, and the oral microbiota. The oral microbiota contributes to both oral and systemic health, and alterations in its composition have been associated with metabolic disorders, including obesity and type 2 diabetes [50]. Furthermore, oral bacteria can influence taste perception [51] and taste bud function through direct interactions with lingual epithelial cells as well as indirectly by modulating local inflammatory and metabolic pathways [52]. In the present study, spheroids were cultured under sterile conditions in the presence of antibiotics to enable characterization of their intrinsic structural and functional properties. Future studies could therefore adapt this model for co-culture with selected commensal or pathogenic oral bacteria to investigate how individual bacterial species or defined bacterial communities regulate taste bud homeostasis, differentiation, and function.

Likewise, incorporating gustatory neurons would further enhance the physiological relevance of this model. Taste bud maintenance critically depends on sensory innervation, as denervation leads to taste bud degeneration, whereas reinnervation restores both taste bud structure and function [53]. Beyond their role in transmitting sensory information, gustatory neurons actively contribute to taste tissue homeostasis by providing trophic signals that regulate stem cell activity, proliferation, differentiation, and regeneration. Several neuron-derived signaling molecules have been implicated in these processes. In particular, Sonic Hedgehog (SHH), expressed by a subset of gustatory neurons, promotes the differentiation of taste cell precursors into mature taste cells [54] and regeneration of taste bud cells [55], although its precise contribution to taste bud homeostasis remains to be fully clarified. In parallel, neuron-derived R-spondins, activate Wnt/β-catenin signaling through LGR5/6-positive stem cells, thereby stimulating progenitor cell proliferation and sustaining taste bud regeneration [49]. Administration of exogenous R-spondin-2 has been shown to prevent taste bud loss following gustatory nerve transection, underscoring its central role in taste bud maintenance; consistent with these trophic properties, R-spondin-1 is commonly included in organoid culture media to support stem cell proliferation and differentiation. Together, these findings identify gustatory neurons as an essential component of the taste stem-cell niche and highlight their role in regulating taste bud renewal and maintenance through multiple signaling pathways. Incorporating gustatory neurons into our spheroid model would therefore provide a more physiologically relevant platform to investigate how neuron-derived trophic signals regulate taste cell differentiation, maintenance, regeneration, and the functional maturation of taste bud cells.

In conclusion, the scaffold-free spheroid model developed in this study provides a reproducible and physiologically relevant 3D system for investigating taste bud cell biology. By combining immortalized CD36^+^ taste bud cells with cells derived from whole circumvallate papillae, we generated multicellular spheroids enriched in lipid-sensing cells while preserving the main taste bud cell populations. The strong upregulation of *CD36* expression, together with the enhanced calcium and serotonin responses to linoleic acid, supports the relevance of this model for studying the cellular and molecular mechanisms of fat taste. At the same time, the observed increased response to monosodium glutamate indicates that these spheroids possibly retain broader taste-cell properties. Overall, this model provides a valuable platform for studying fat taste in a more physiologically relevant context while reducing reliance on animal experimentation.

## 4. Materials and Methods

### 4.1. Generation of Lingual Spheroids (3D) and Monolayer Cells (2D)

Two cell lines were used in this study: whole circumvallate papillae-derived taste bud cells and CD36-positive taste bud cells. Taste bud cells (TBCs) from C57Bl/6 mice were initially isolated from lingual papillae by enzymatic dissociation of the epithelium using an elastase/dispase mixture (2 mg/mL each), followed by digestion with EDTA (2 mM), elastase (1.2 mg/mL), type I collagenase (0.6 mg/mL), and trypsin inhibitor (0.6 mg/mL) to obtain a single-cell suspension. These cells were collected by centrifugation and immortalized using mouse telomerase reverse transcriptase (mTERT) retroviral transfection, generating stable taste bud cell lines as described in Benmouna et al. [56]. These cells have been deposited at the Collection Nationale de Cultures de Microorganismes (CNCM, Institut Pasteur, Paris, France) under file no. 3907 (5 February 2020). CD36-positive cells were subsequently purified from these total immortalized TBCs using magnetic-activated cell sorting (MACS), as described by El-Yassimi et al. [16]. Briefly, cells were labelled with an anti-CD36 antibody conjugated to phycoerythrin, followed by incubation with anti-phycoerythrin microbeads, and separated using magnetic columns to isolate CD36-positive cells, which were then cultured for further experiments.

For spheroids and monolayer cultures, these two cell lines—whole circumvallate papillae-derived cells and CD36-positive taste bud cells were mixed at a 1:1 ratio, with a total of 5000 cells seeded in 100 µL of medium per well in 96-well plates. For spheroid formation, cells were plated in ultra-low-adherence 96-well plates (Thermo Fisher Scientific, Roskilde, Denmark) and centrifuged (2000 rpm for 5 min) 24 h after seeding. For monolayer cultures, standard 96-well plates were used.

Monolayer cultures were maintained in conditioned IMDM medium (20% Keratinocyte Basal Medium, 10% foetal bovine serum, 1% penicillin/streptomycin/amphotericin B). Spheroids were cultured in conditioned DMEM/F12 medium supplemented with 100 ng/mL Noggin, 50 ng/mL epidermal growth factor (EGF), 1% N2 supplement, 2% B27 supplement, 200 ng/mL R-spondin 1, 10 µM Y-27632 dihydrochloride, 1% penicillin/streptomycin/amphotericin B, and 1:1000 Primocin, corresponding to organoid culture conditions for taste bud cells, as described in previous studies [24,25,26,27,28]. All spheroids used in this study were generated after 7 days of culture.

### 4.2. Cryo-Scanning Electron Microscopy

Spheroids were fixed in a solution containing 4% paraformaldehyde and 2.5% glutaraldehyde in 0.1 M PHEM buffer for 25 min. Samples were then washed 3 × 5 min in the same buffer and stained with 0.05% toluidine blue in water.

Spheroids were subsequently mounted onto previously glow-discharged silica coupons, rapidly frozen at −150 °C using a Quorum PP3010 cryo-stage (Quorum, Laughton, UK), and observed either without sublimation or after a 1 min sublimation step at −90 °C (as indicated on the images). Samples were imaged with or without platinum coating (10 s at 10 mA, as specified on the images).

Observations were performed using a TESCAN Clara scanning electron microscope (SEM) (TESCAN, Brno, Czech Republic) at the Dimacell platform (INRAE/Université Bourgogne Europe, Dijon, France).

### 4.3. Transmission Electron Microscopy

Spheroids were fixed with 4% paraformaldehyde and 2.5% glutaraldehyde in 0.1 M PHEM buffer for 25 min. They were washed 3 × 5 min with the same buffer then stained with 0.05% toluidine blue in water and embedded in low-melting point agarose. Samples were post-fixed with 0.5% osmium tetroxide in water for 45 min in the dark at +4 °C. They were washed with PHEM buffer for 5 min at +4 °C, then in water for 5 min. Samples were dehydrated with graded ethanol and propylene oxide and then embedded in EMBed-812 resin. Resin Polymerization was performed at 68 °C for 48 h. Ultrathin sections (80 nm) were cut on a Reichert Ultracut E (Leica Microsystems SAS, Nanterre, France) and were collected onto copper grids. The grids were contrasted with uranyl acetate and lead citrate and observed on a Hitachi HT7800 TEM (Hitachi High-Tech Corporation, Tokyo, Japan) operating at 100 kV and equipped with an AMT camera NanoSprint 43 (Advanced Microscopy Techniques, Woburn, MA, USA) at the Dimacell platform (INRae/Université Bourgogne Europe, Dijon, France).

### 4.4. Haematoxylin and Eosin Staining

Histological experiments were performed at the ImaFlow Facility (US58 BioSanD, Université Bourgogne Europe, Dijon, France). 7-day spheroids were collected and fixed in 10% buffered formaldehyde for 20 min, stored in 70% ethanol, washed with PBS and then put in Histogel. Spheroids were then dehydrated in graded alcohols, cleared in xylene, and embedded in paraffin on Leica ASP300 Tissue processor (Leica, Wetzlar, Germany). 5 µm-thick slices were made on a Leica rotary microtome (Histocore Autocut R, Leica, Wetzlar, Germany) and deposited on coated glass slides to achieve histological analysis. After deparaffinization and rehydration steps, sections were stained on Leica AutoStainer XL ST5010 (Leica, Wetzlar, Germany) to achieve a haematoxylin–eosin stain with Harris Haematoxylin (Leica #3801522E) and Eosin Y alcoholic (Leica #3801602E) solutions. Stained slides were dehydrated and mounted in organic mounting medium Coverquick 4000 (VWR #05547539).

### 4.5. Immunohistochemistry

Tissue sections (5 µM) on slides were deparaffinized in xylene and rehydrated in decreasing concentrations of ethanol. For the antigen retrieving, rehydrated tissue sections were incubated in Citrate buffer pH 6 at 95 °C for 20 min (ImaFlow Facility, US58 BioSanD, Université Bourgogne Europe, Dijon, France). Slides were washed twice with TBS 1X for 5 min, then saturated with TBS- 10% FBS for 1 h at room temperature. Slides were then incubated with primary antibody solutions in 1/100 in TBS-1% FBS overnight at +4 °C: anti-GLAST (PA5-72895, Invitrogen, Carlsbad, CA, USA); anti-α-gustducin (NBP1-20926, Novus Biological, Abingdon, UK); anti-SNAP-25 (NBP1-00858, Novus Biological, Abingdon, UK); anti-CD36 (NB400-145, Novus Biological, Abingdon, UK). Slides were washed, then incubated for 2 h30 min in the dark with the corresponding 1/1000 diluted secondary antibody coupled to Alexa Fluor-488 (green) or Alexa Fluor-568 (red): Alexa Fluor 488 donkey anti-goat (A32814, Invitrogen, Carlsbad, CA, USA); Alexa Fluor 568 donkey anti-rabbit (A10042, Invitrogen, Carlsbad, CA, USA), Alexa Fluor 568 goat anti-mouse (A10042, Invitrogen, Carlsbad, CA, USA). Slides were coated with a 1/750 solution of HOECSHT (1 mg/mL; 62249, Thermo Fisher Scientific, Roskilde, Denmark) in TBS for 5 min in the dark, then mounted with Prolong Diamond Anti-fade Mountant (P3691, Invitrogen, Carlsbad, CA, USA). Slides were observed using Leica SP8, an inversed confocal fluorescent microscope at the Dimacell platform (INRAE/Université Bourgogne Europe, Dijon, France) and images were analyzed with LAS X Office software version 1.4.7.28982. Cell type quantification was performed with QuPath software version 0.7.0 according to Lumanto and colleagues [57].

### 4.6. Quantitative Real-Time Polymerase Chain Reaction

Six spheroids, one from each of six wells, and cells collected from six wells of 96-well plates were used for RNA extraction from cells cultured under 3D and 2D conditions, respectively. Total RNA was extracted using TRIzol reagent (Invitrogen Life Technologies, Bleiswijk, The Netherlands). Lysates were homogenized with syringes. RNA quantification was performed by spectrophotometry (NanoPhotometer^®^ N60/N50, Implen, Munich, Germany). 0.5 μg of total RNA was reverse transcribed with High-Capacity cDNA Reverse Transcription Kit (4368814, Applied Biosystems, Foster City, CA, USA) according to the manufacturer’s recommendations. cDNA was amplified using a Step OnePlus device (Life Technologies, Saint-Aubin, France) with the use of PowerSYBR Green PCR Master Mix (4367659, Applied Biosystems, Foster City, CA, USA). Three independent experiments were performed, each in at least triplicate. Quantitative real-time polymerase chain reaction was performed using the following protocol: holding stage, 50 °C for 2 min, 95 °C for 10 min; 40 cycles at 95 °C for 15 s, 60 °C for 1 min (except for *VMAT-2*, *T1R2* and *Sox2*, where it was 52 °C, 62 °C and 66 °C respectively). mRNA expression was normalized to housekeeping mRNA GAPDH. Relative mRNA quantification in the different samples was determined by the comparative 2^−ΔΔCT^ method. Outliers were removed and Mann–Whitney tests were performed with GraphPad Prism 10 software. Primers used for qPCR are shown in Table 1.

### 4.7. Calcium Signaling Measurement

2D- and 3D-cultured cells were cultured in their respective media, and both were dissociated by Accutase (00-4555-56, Invitrogen, Carlsbad, CA, USA) for 10 min at 37 °C and 5% CO_2_. Dissociated cells were then seeded in equal number per well onto 96-well WillCo-dish plates in RPMI 1640 and 1% Penicillin/Streptomycin/Amphoterin overnight. Prior to the experiments, cells were washed with PBS and incubated at 37°C for 45 min with 1 µM Fluo4/AM, fluorescent calcium-sensitive probe in RPMI 1640 with 1% Penicillin/Streptomycin/Amphoterin B prior to experiments in loading buffer (110 mM NaCl; 5.5 mM KCl; 25 mM NaHCO_3_; 0.8 mM MgCl_2_; 0.4 mM KH_2_PO_4_; 0.33 mM Na_2_HPO_4_; 20 mM HEPES; 1.2 mM, CaCl_2_; pH: 7.4). Fluorescence was monitored using a Nikon microscope (TiU) equipped with an EM-CCD (Luca-S) camera for real-time recording of digital images and an S-fluor 40× oil immersion objective (Nikon, Tokyo, Japan). A defined number of regions of interest (ROI) were manually positioned using auto-detection and then polygon functions in NIS-Elements AR software version 3.2. Changes in fluorescence were calculated as ΔF/F_0_ = (F − F_0_)/F, F_0_, determined as the first fluorescence measure. (ΔF/F_0_)_max_ is the maximum of fluorescence intensity during the recording. All tested molecules were added in small volumes with no interruption in recordings. Linoleic acid (Sigma-Aldrich L1376-5g, Sigma-Aldrich Chimie, Saint-Quentin-Fallavier, France) and monosodium glutamate (Sigma-Aldrich G1626-1kg, Sigma-Aldrich Chimie, Saint-Quentin-Fallavier, France) were added in the WillCo-dish wells after 2 min of recording. Data were obtained from at least two independent experiments, each performed in triplicate, and statistical analysis was performed using nested *t*-tests.

### 4.8. Serotonin Secretion Quantification

7 day 3D-cultured cells maintained in conditioned DMEM/F12 were centrifuged at 2000 rpm for 5 min, washed with PBS, and centrifuged again at 2000 rpm for 5 min. Six spheroids, one from each of six wells, were then pooled into a single sample. 3-day-cultured 2D cells maintained in conditioned IMDM were used as controls. Plates were incubated for 6 h 30 with RPMI 1640 with 1% Penicillin/Streptomycin/Amphoterin B. 2D- and 3D-cultured cells were then stimulated with vehicle solution or 100µM of linoleic acid for 10 min, at 37 °C and 5% CO_2_. After 10 min, supernatants of stimulated cells were collected, frozen in liquid nitrogen and stored at −80 °C and cells were lysed with RIPA 2×, with 1/100 protease inhibitor cocktail (Roche 1187358001, Sigma-Aldrich Chimie, Saint-Quentin-Fallavier, France) and 1/100 phosphatase inhibitor cocktail (Sigma-Aldrich P5726, Sigma-Aldrich Chimie, Saint-Quentin-Fallavier, France). Experiments were performed in duplicates in at least three independent experiments. Proteins were quantified by Pierce™ BCA Protein Assay Kit (ThermoFisher A55865, Thermo Fisher Scientific, Roskilde, Denmark). Serotonin was quantified in supernatants by Serotonin ELISA kit (Serotonin ELISA kit, ADI-900-175, Enzo Life Sciences, Farmingdale, NJ, USA). According to the manufacturer’s specifications, the assay sensitivity was 0.293 ng/mL, while the lowest standard included in the calibration curve was 0.49 ng/mL. The intra-assay coefficients of variation (CVs) were 4.2%, 5.8%, and 11.0% at serotonin concentrations of 346.1, 53.8, and 13.5 ng/mL, respectively, whereas the corresponding inter-assay CVs were 16.2%, 18.4%, and 12.7%. Importantly, all OD values obtained for our samples were within the range of the standard calibration curve, confirming that the serotonin measurements were performed within the assay’s analytical range.

For each well, serotonin quantity in supernatant was divided total protein quantity after cell lysis (pg/µg of protein). Two-way ANOVA with Tukey’s multiple comparisons test was performed with GraphPad Prism 10 software.

### 4.9. Live/Dead Assays

Spheroids were cultured for 7 days. Medium was changed with 1% Cyto3D^®^ Live-Dead Assay Kit (BM01, The Well Bioscience, Monmouth Junction, NJ, USA) and spheroids were incubated for 10 min. Spheroids were incubated with 10% DMSO for 10 min prior to the assay as a negative control. Live and dead cells are labeled green and red respectively. Spheroids were washed 3 × 5 min in PBS, collected and fixed in 10% buffered formaldehyde for 20 min, stored in 70% ethanol, washed with PBS and then put in Histogel. Spheroids were then dehydrated in graded alcohols, cleared in xylene, and embedded in paraffin on Leica ASP300 Tissue processor (Leica, Wetzlar, Germany). 5 µm-thick slices were made on a Leica rotary microtome (Histocore Autocut R, Leica, Wetzlar, Germany) and deposited on coated glass slides to achieve histological analysis. Slides were observed with Axio Imager M2 epifluorescence microscope (Zeiss, Oberkochen, Germany). Live/dead quantification was performed with QuPath software version 0.7.0 according to Lumanto and colleagues [57].

## Figures and Tables

**Figure 1:**
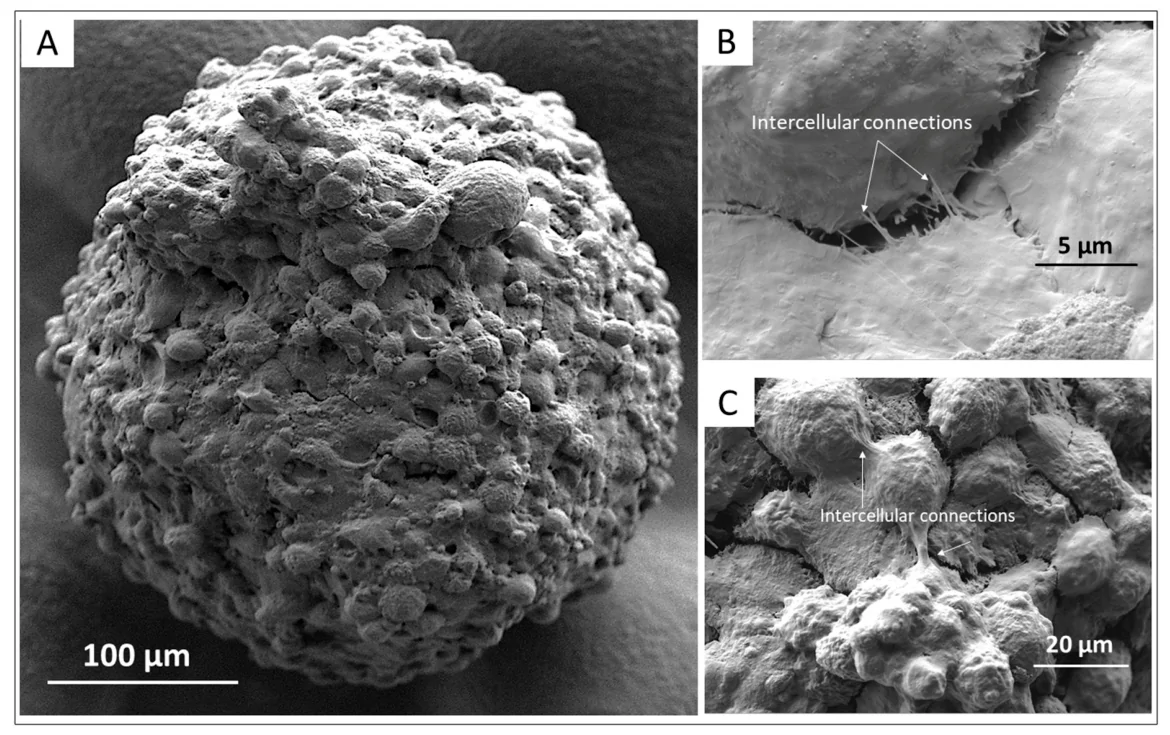
Cryo-scanning electron microscopy of 7-day taste bud cell spheroids. Taste bud cell spheroids were generated by co-culturing whole circumvallate papillae-derived cells and CD36^+^ taste bud cells (1:1) under ultra-low-adherence conditions for 7 days, then fixed and processed for cryo-scanning electron microscopy. (**A**) Whole spheroid showing a compact spherical organization. (**B**) Higher-magnification view of the spheroid surface highlighting intercellular connections between adjacent cells. (**C**) Higher-magnification view showing the rough cell surface and surface-associated granules. Scale bars: 100 µm (**A**), 20 µm (**B**), 5 µm (**C**).

**Figure 2:**
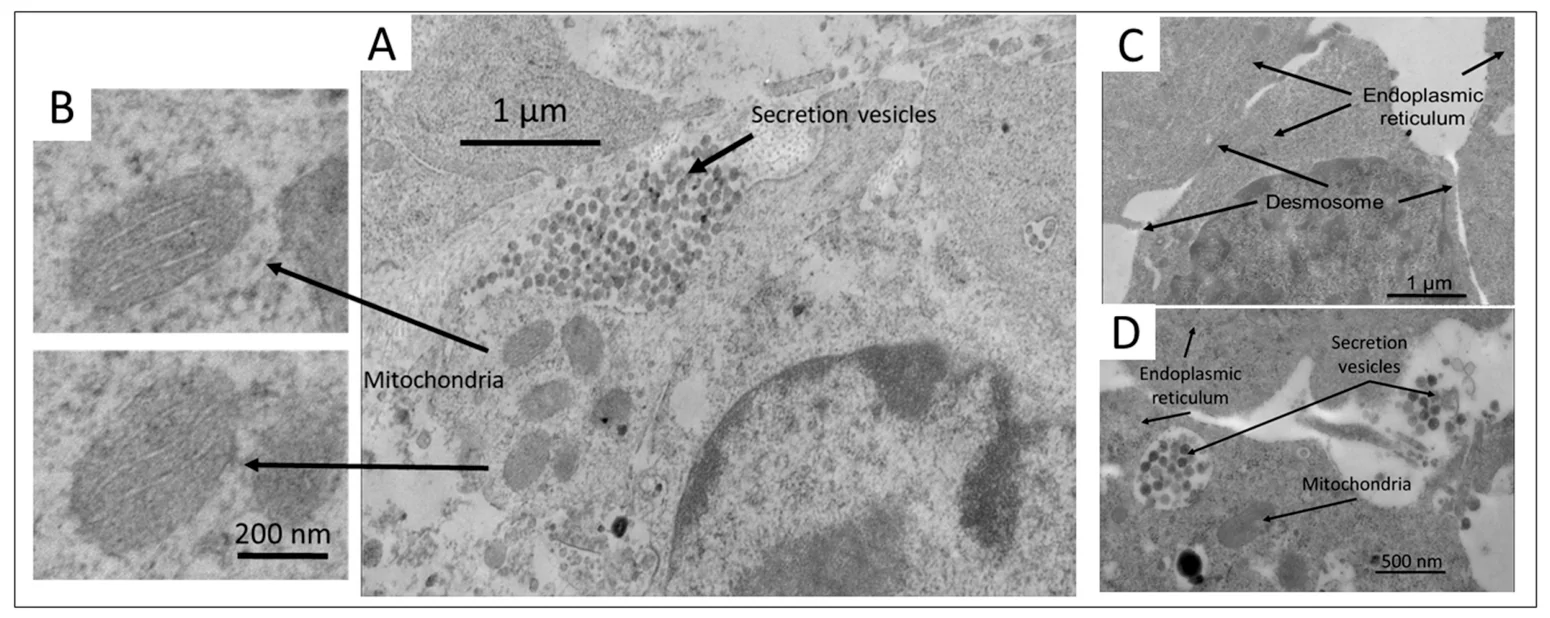
Transmission electron microscopy of 7-day taste bud cell spheroids. Taste bud cell spheroids were generated by co-culturing whole circumvallate papillae-derived cells and CD36^+^ taste bud cells (1:1) under ultra-low-adherence conditions for 7 days, then fixed, embedded in resin, sectioned, and analyzed by transmission electron microscopy. (**A**) Representative cell showing numerous mitochondria and secretory vesicles. (**B**) Higher-magnification views of mitochondria from panel (**A**). (**C**) Adjacent cells showing abundant endoplasmic reticulum and desmosomal contacts. (**D**) Representative view of two neighboring cells containing mitochondria and secretory vesicles, including vesicles observed in the intercellular space. Scale bars: 1 µm (**A**,**C**), 500 nm (**D**).

**Figure 3:**
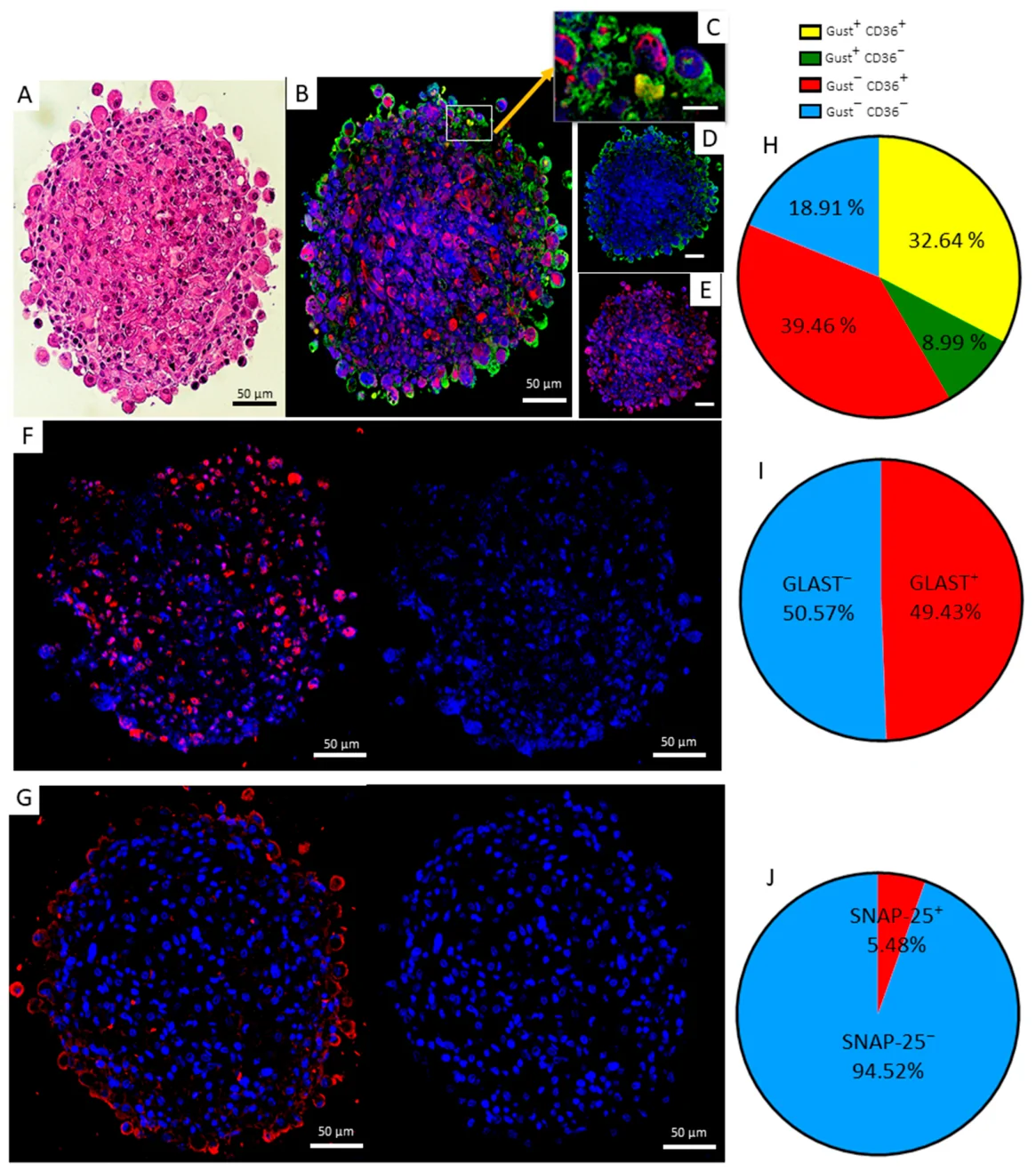
Histological and immunofluorescence characterization of 7-day taste bud cell spheroids. Taste bud cell spheroids were generated by co-culturing whole taste papilla-derived cells and CD36^+^ taste bud cells (1:1) under ultra-low-adherence conditions for 7 days, then fixed, paraffin-embedded, sectioned, and analyzed by histology or immunofluorescence. (**A**) Hematoxylin and eosin staining showing the overall spheroid organization, with nuclei in purple and cytoplasm in pink. (**B**) Double immunofluorescence staining for α-gustducin (green) and CD36 (red), with DAPI nuclear counterstaining (blue). (**C**) Higher-magnification view showing co-localization of α-gustducin and CD36 in a subset of cells. Scale bar = 10 µm (**D**) Immunofluorescence staining for α-gustducin (green) with DAPI (blue). Scale bar = 50 µm (**E**) Immunofluorescence staining for CD36 (red) with DAPI (blue). Scale bar = 50 µm (**F**) Immunofluorescence staining for GLAST (red), a type I taste cell marker, with DAPI (blue). (**G**) Immunofluorescence staining for SNAP-25 (red), a type III taste cell marker, with DAPI (blue). Scale bars: 50 µm unless otherwise indicated. (**H**–**J**) Quantitative analysis of cell types was performed using QuPath software.

**Figure 4:**
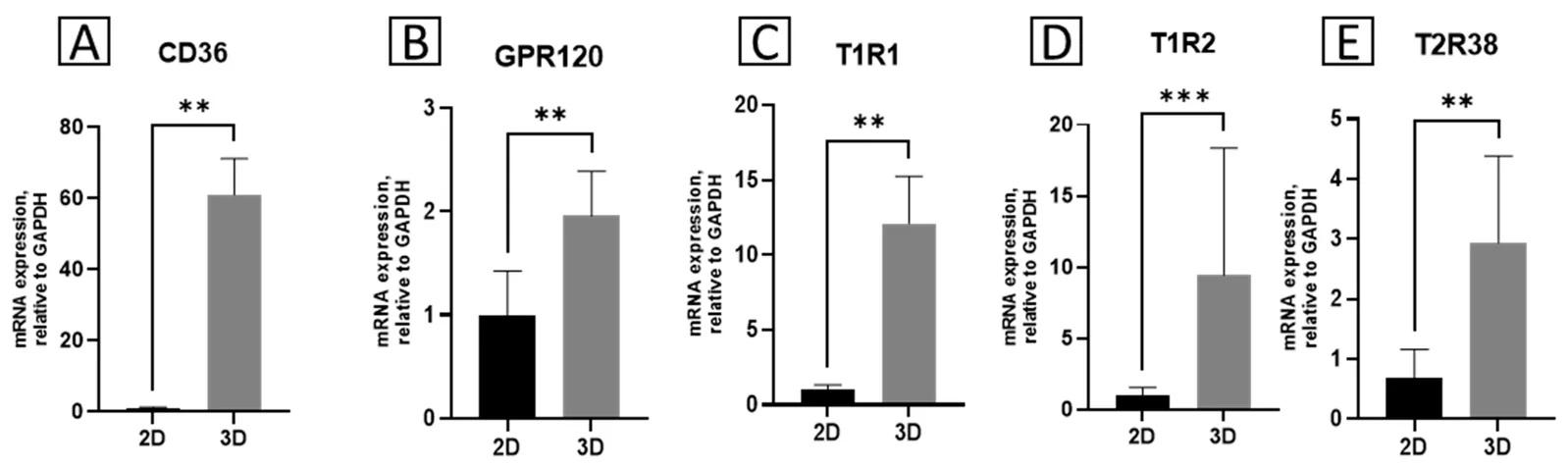
Taste receptors gene expression profile of 2D- and 3D-cultured taste bud cells. mRNA expression levels were analyzed by qRT-PCR in cells cultured under 2D or 3D conditions. Expression of taste receptor genes, including (**A**) *CD36*, long-chain fatty acid receptor; (**B**) *GPR120*, long-chain fatty acid receptor; (**C**) *T1R1*, umami receptor subunit; (**D**) *T1R2*, sweet receptor subunit; (**E**) *T2R38*, bitter taste receptor. Gene expression was normalized to *GAPDH* and expressed relative to 2D-cultured cells using the 2^−ΔΔCT^ method. Data were obtained from at least three independent experiments, each performed in triplicate, and are presented as mean ± SD. Statistical analysis was performed using Mann–Whitney tests. ** *p* < 0.01, *** *p* < 0.001.

**Figure 5:**
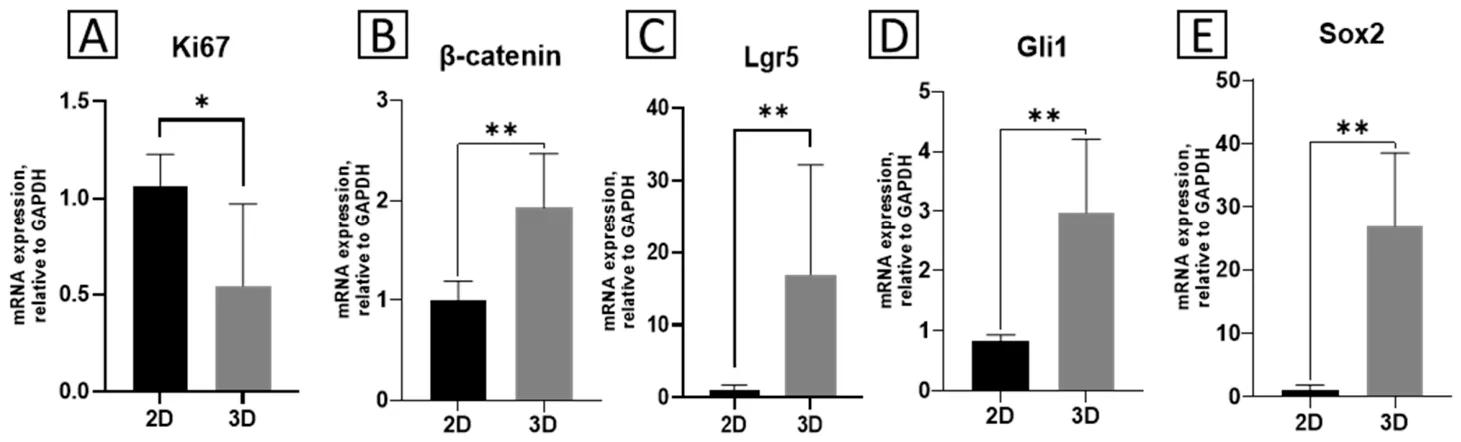
Proliferation and differentiation markers gene expression profile of 2D- and 3D-cultured taste bud cells. mRNA expression levels were analyzed by qRT-PCR in cells cultured under 2D or 3D conditions. (**A**) Expression of the proliferation marker *Ki67*. (**B**) Expression of *β-catenin* and (**C**) *Lgr5*, two genes associated with the Wnt signaling pathway. (**D**) Expression of *Gli1* and (**E**) *Sox2*, two genes associated with the Sonic Hedgehog (Shh) pathway. Gene expression was normalized to *GAPDH* and expressed relative to 2D-cultured cells using the 2^−ΔΔCT^ method. Data were obtained from at least three independent experiments, each performed in triplicate, and are presented as mean ± SD. Statistical analysis was performed using Mann–Whitney tests. * *p* < 0.05, ** *p* < 0.01.

**Figure 6:**
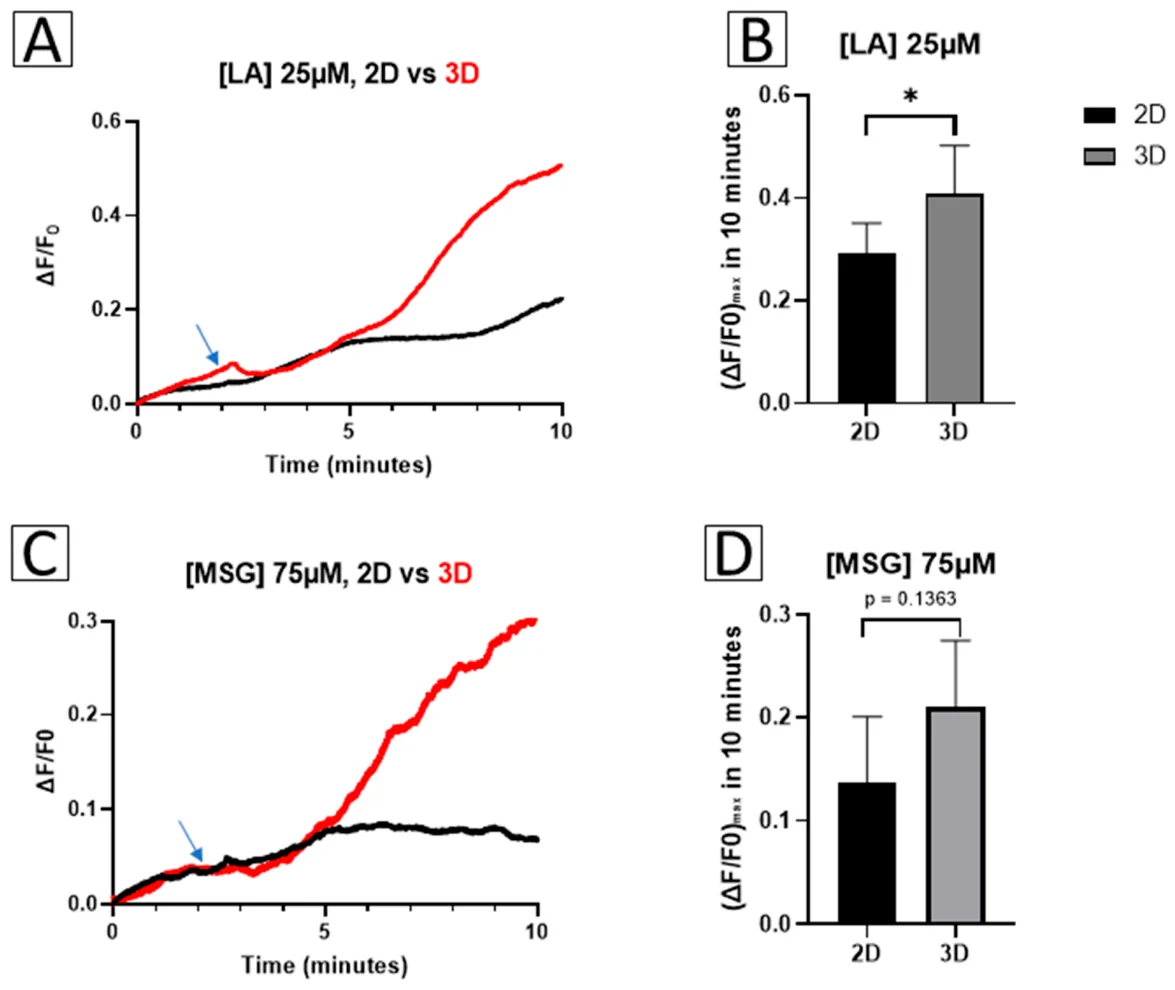
Cells derived from 3D spheroids display enhanced calcium responses to linoleic acid and monosodium glutamate. Cells cultured under 2D conditions or dissociated from 7-day spheroids were loaded with Fluo-4 AM and intracellular calcium responses were monitored by live fluorescence imaging. Tastants were added after 2 min of recording, as indicated by the blue arrow. (**A**) Representative traces of intracellular calcium responses induced by 25 µM linoleic acid. (**B**) Quantification of the maximal fluorescence variation (ΔF/F_0_)_max_ in response to linoleic acid. (**C**) Representative traces of intracellular calcium responses induced by 75 µM monosodium glutamate (MSG). (**D**) Quantification of the maximal fluorescence variation (ΔF/F_0_)_max_ in response to MSG. Data were obtained from at least two independent experiments, each performed in triplicate, and are presented as mean ± SD. Statistical analysis was performed using nested *t*-tests. * *p* < 0.05.

**Figure 7:**
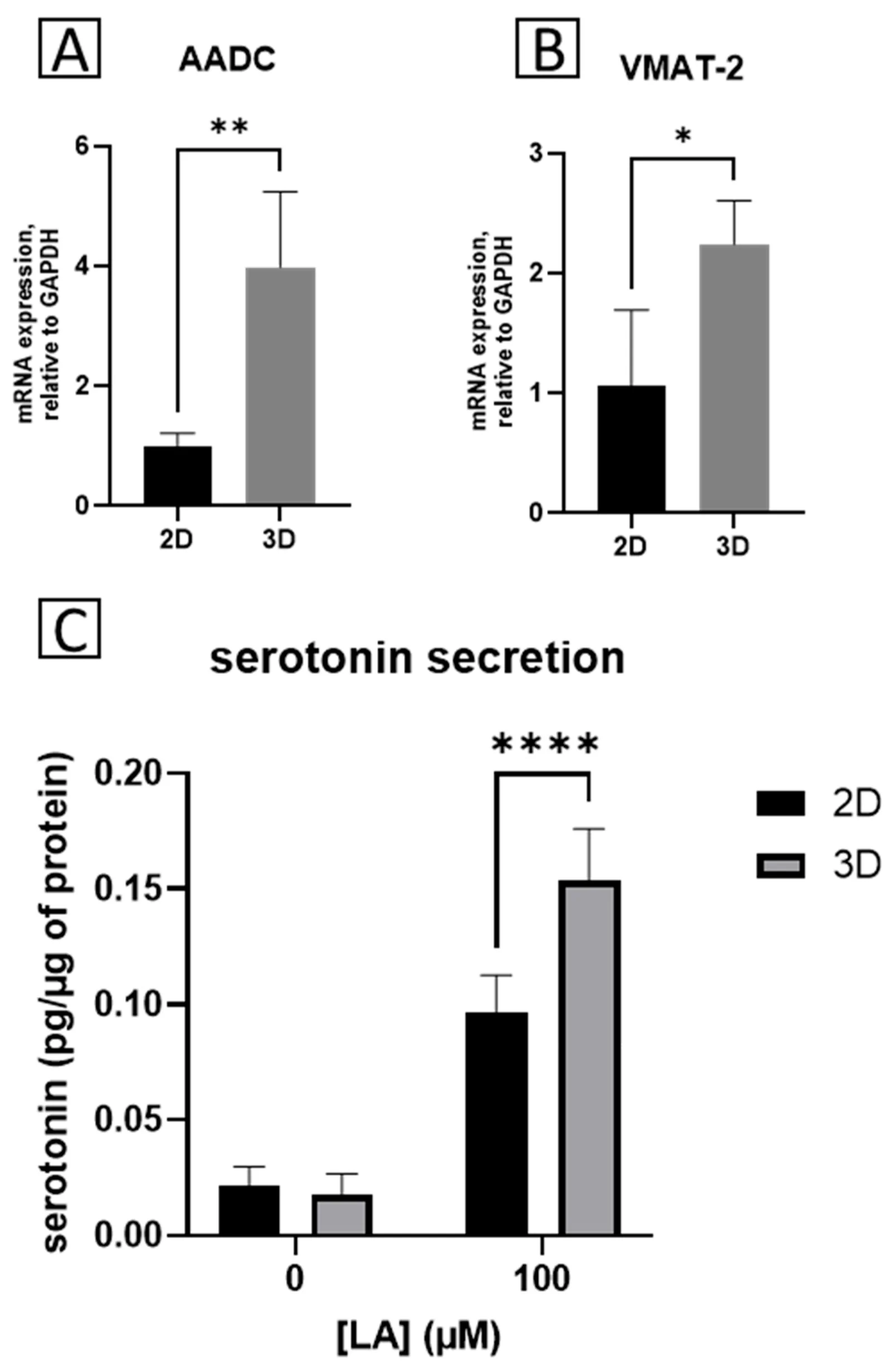
Cells derived from 3D spheroids show increased expression of serotonin-related genes and enhanced serotonin release in response to linoleic acid. mRNA expression of (**A**) *AADC* (aromatic L-amino acid decarboxylase) and (**B**) *VMAT-2* (vesicular monoamine transporter 2) was analyzed by qRT-PCR in 2D- and 3D-cultured taste bud cells. Gene expression was normalized to *GAPDH* and expressed relative to 2D-cultured cells using the 2^−ΔΔCT^ method. Statistical analysis was performed using Mann–Whitney tests. (**C**) Serotonin secretion was quantified by ELISA after 10 min stimulation with control solution or 100 µM of linoleic acid (LA). Serotonin levels were normalized to total cellular protein content and expressed as pg/µg protein. Data were obtained from at least three independent experiments, each performed in duplicate, and are presented as mean ± SD. Statistical analysis was performed using a two-way ANOVA with Tukey’s multiple comparisons test. * *p* < 0.05, ** *p* < 0.01, **** *p* < 0.0001.

**Table 1:** Primer Sequences for Polymerase Chain Reaction.

Genes	Forward Primer	Reverse Primer
*AADC*	5′-TCCTGCTGTTCTTTTGACAATCTC-3′	5′-GCGTAAGCAGCGTCAATGTG-3′
*β-catenin*	5′-CCCAGTCCTTCACGCAAGAG-3′	5′-CATCTAGCGTCTCAGGGAACA-3′
*CD36*	5′-CCGAACACAGCGTAGATAGAC-3′	5′-GGCCAAGCTATTGCGACATG-3′
*GAPDH*	5′-AAATGGTGAAGGTCGGTGTG-3′	5′-TCGTTGATGGCAACAATCTC-3′
*Gli1*	5′-GGAAGTCCTATTCACGCCTTGA-3′	5′-CAACCTTCTTGCTCACACATGTAAG-3′
*GPR120*	5′-GTGCCGGGACTGGTCATTGTG-3′	5′-TTGTTGGGACACTCGGATCTGG-3′
*Ki67*	5′-TCTGATGTTAGGTGTTTGAG-3′	5′-CACTTTTCTGGTAACTTCTTG-3′
*LGR5*	5′-AGCATGCTTCTGGCAAGATGTTC-3′	5′-GACTTAACGCCCTGCGTTTGA-3′
*Sox2*	5′-CCAGCGCATGGACAGCTA-3′	5′-GCTGCTCCTGCATCATGCT-3′
*T1R1*	5′-CCTGACTGGCAAAGTGTGGA-3′	5′-TCCATGTCGTGAAAGCGTGA-3′
*T1R2*	5′-CCGCCAGGCTTTCTTCACC-3′	5′-AGACGCACACAATCTGGAAGG-3′
*T2R38*	5′-CTGTGACATCGCACTGCTGT-3′	5′-AATCTTGGAGCAGTAGAGGA-3′
*VMAT-2*	5′-GCTACCTGTACAGCATTAAGCACG-3′	5′-CCAACTCCAAAGTTGGGAGCG-3′

## Data Availability

The original contributions presented in this study are included in the article/Supplementary Materials. Further inquiries can be directed to the corresponding author.

## Supplementary Materials

The following supporting information can be downloaded at https://www.mdpi.com/article/10.3390/ijms27198545/s1.

## Abbreviations

The following abbreviations are used in this manuscript:

2D	Two dimensions
3D	Three dimensions
CD36	Cluster of Differentiation CD36
CVP	Circumvallate papillae
FFAR	Free Fatty Acid Receptor
LA	Linoleic Acid
LCFA	Long-Chain Fatty Acids
MSG	Monosodium Gultamate
TBCs	Taste Bud Cells
