# Supplementary material for: Establishment of a Three-Dimensional In Vitro Model of Taste Bud Cells for Fat Taste Investigation
Source: Int J Mol Sci. 2026 Sep 24;27(19):8545. doi: 10.3390/ijms27198545 (PMC13665549; doi:10.3390/ijms27198545)
Supplement: Supplementary file 1 [file ijms-27-08545-s001.zip › ijms-4505252-supplementary.pdf]

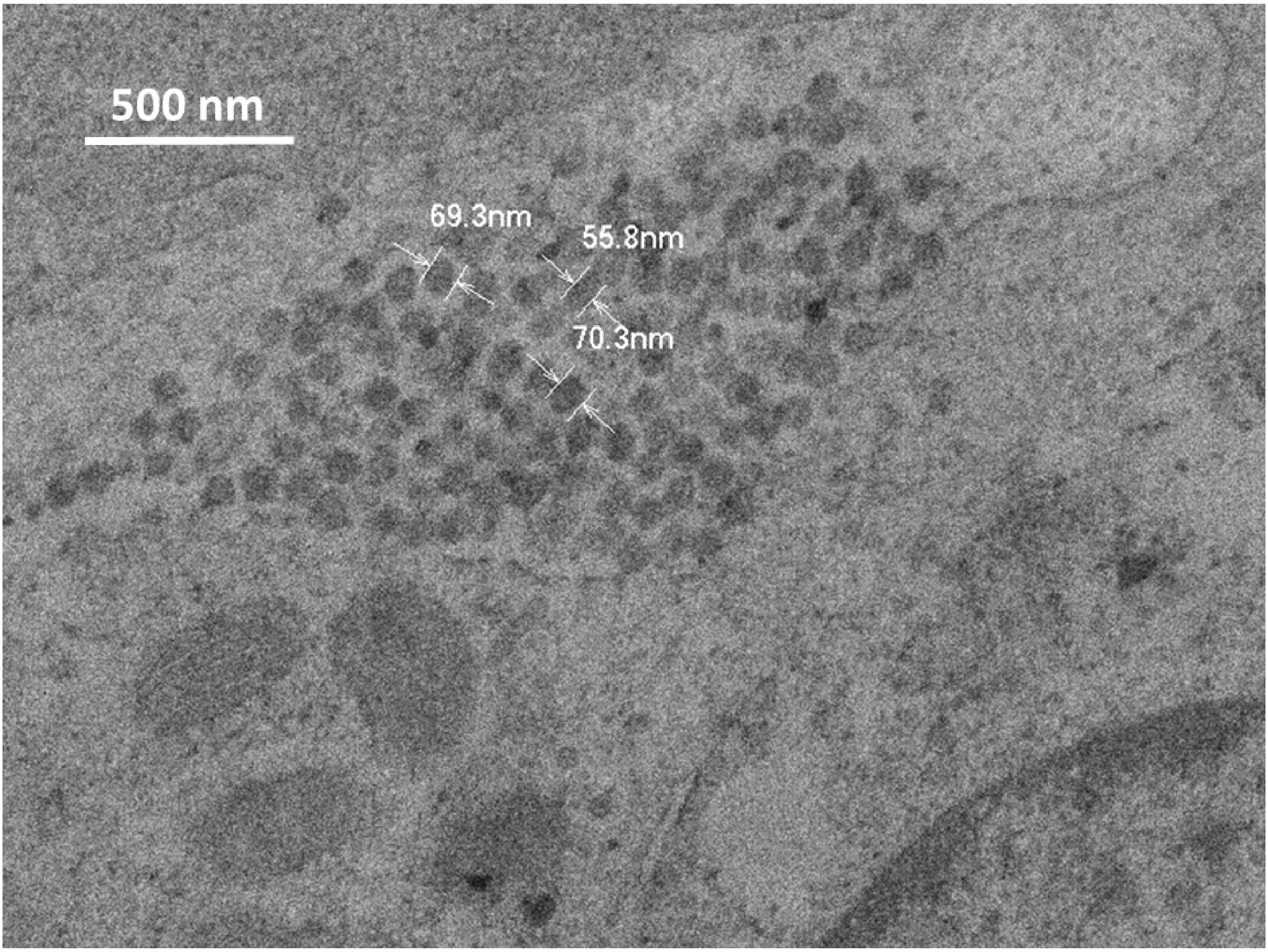


**Supplementary Figure S1**

Measurements of vesicles diameter of Figure 1A. Taste bud cell spheroids were generated by co-culturing whole circumvallate papillae-derived cells and CD36⁺ taste bud cells (1:1) under ultra-low-adherence conditions for 7 days, then fixed, embedded in resin, sectioned, and analyzed by transmission electron microscopy.

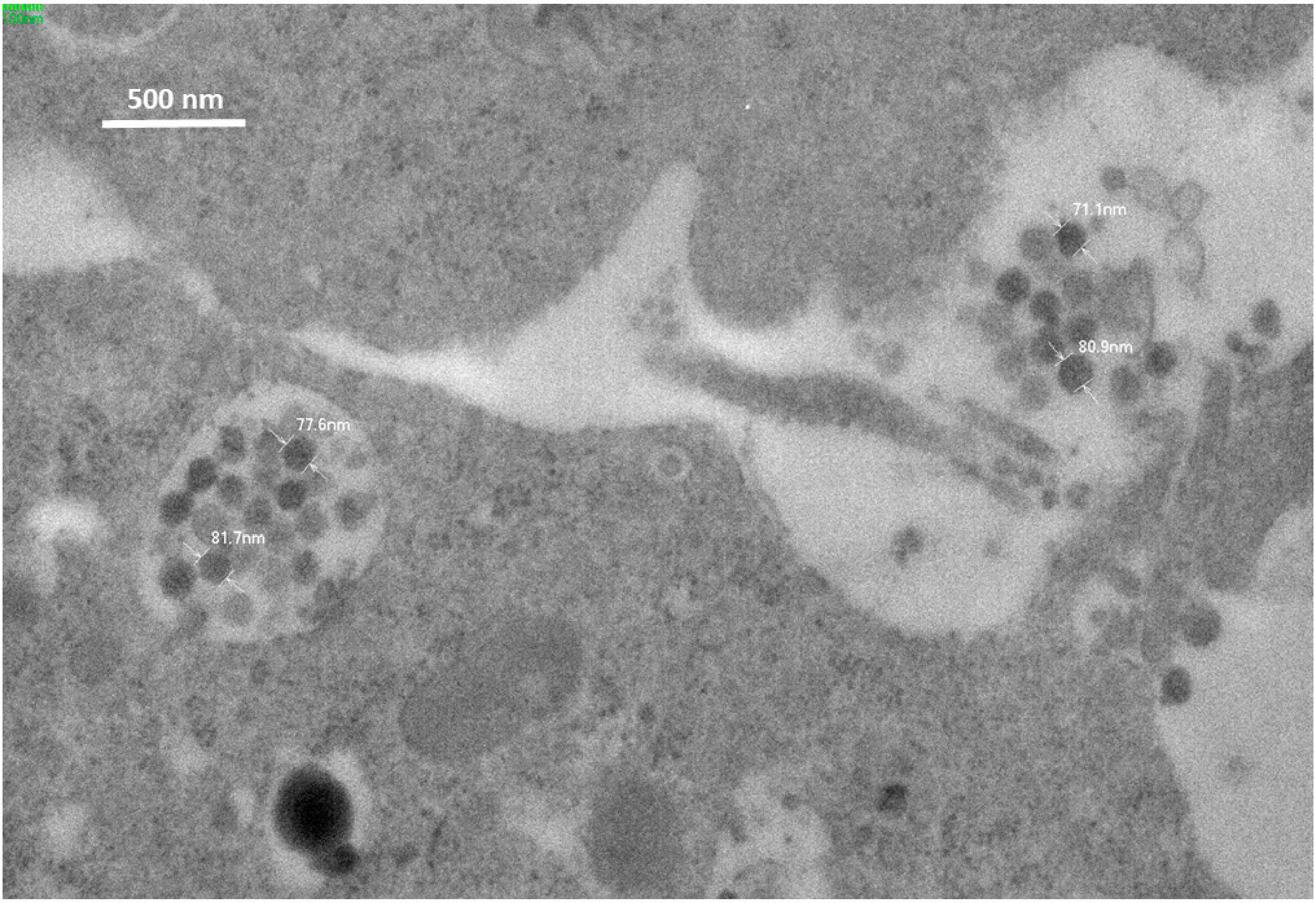


**Supplementary Figure S2**

Measurements of vesicles diameter of Figure 1D. Taste bud cell spheroids were generated by co-culturing whole circumvallate papillae-derived cells and CD36⁺ taste bud cells (1:1) under ultra-low-adherence conditions for 7 days, then fixed, embedded in resin, sectioned, and analyzed by transmission electron microscopy.

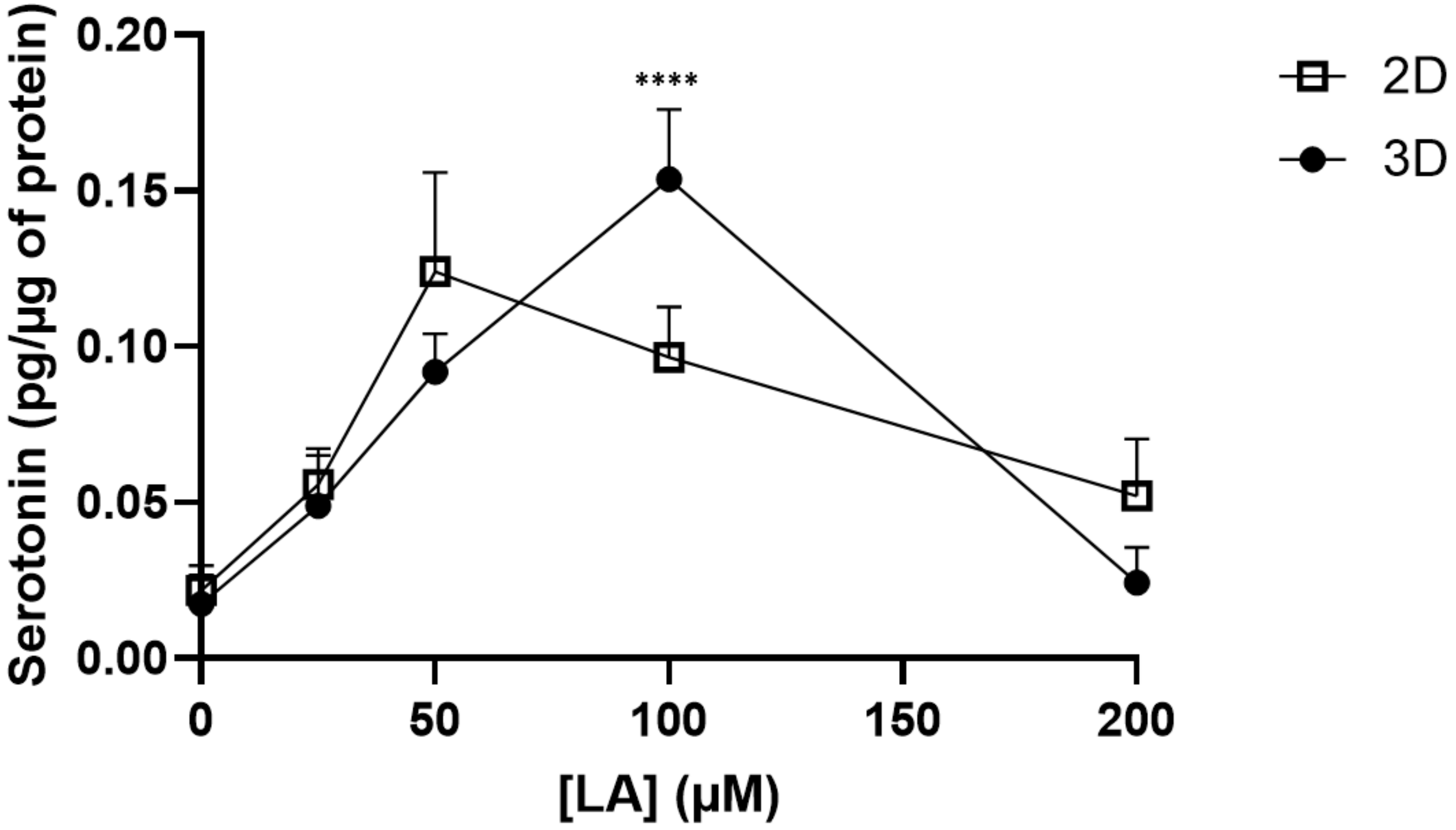


**Supplementary Figure S3**

Serotonin secretion with different linoleic acid (LA) concentration. Serotonin was quantified by ELISA after 10 min stimulation with control solution, 25µM, 50µM, 100 µM or 200 µM of linoleic acid. Serotonin levels were normalized to total cellular protein content and expressed as pg/µg protein. Data were obtained from at least three independent experiments, each performed in duplicate, and are presented as mean ± SD. Statistical analysis was performed using a two-way ANOVA with Tukey's multiple comparisons test. **** $p < 0.0001$.

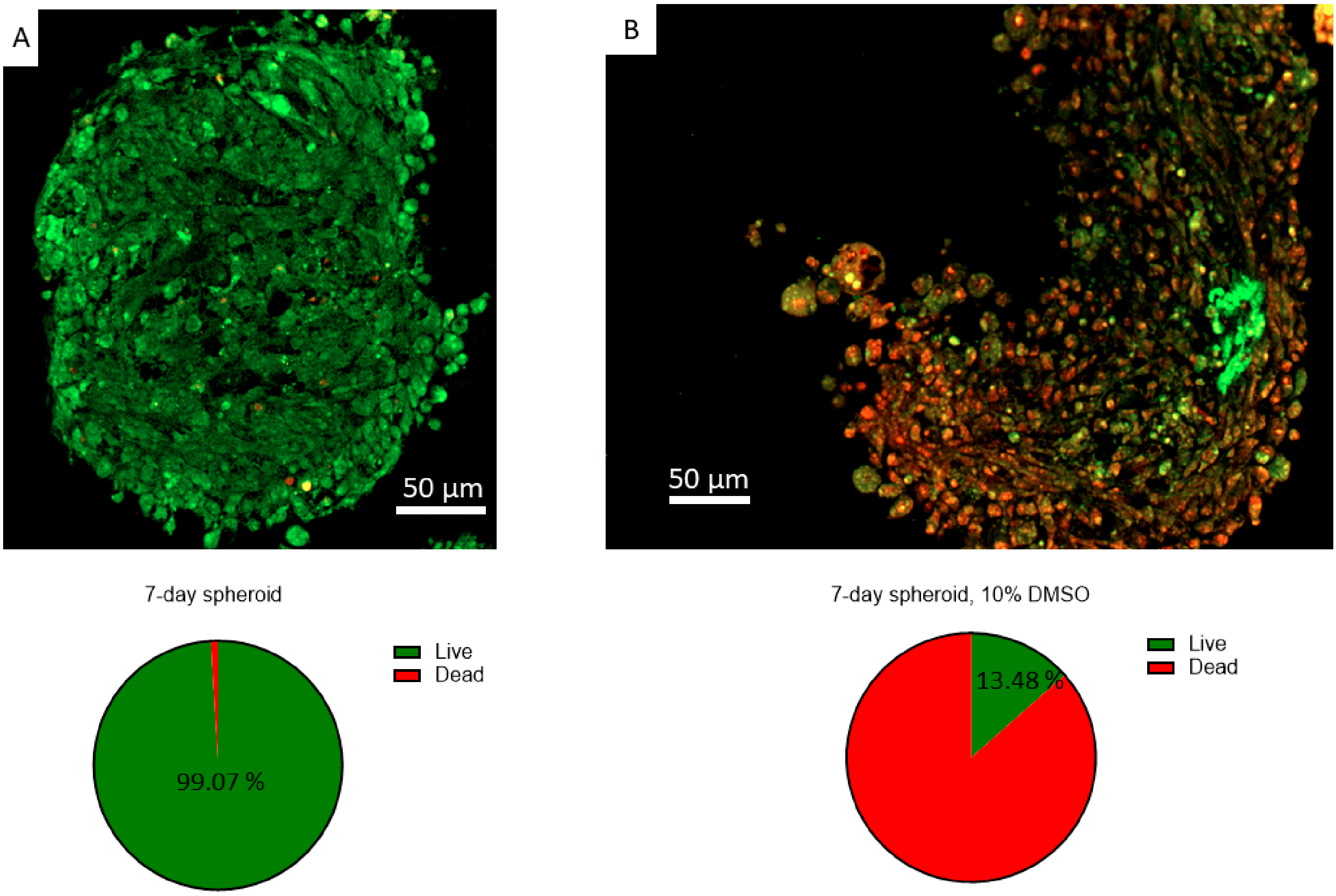


**Supplementary Figure S4**

Viability assay after 7 days of culture. Taste bud spheroids were cultured for 7 days in completed DMEM/F12. Medium was changed with 1% Cyto3D® Live-Dead Assay Kit and spheroids were incubated for 10 minutes (A). Spheroids were incubated with 10% DMSO for 10 minutes as a negative control prior to the assay (B). Live cells are green and dead cells are red. Spheroids were washed 3 x 5minutes in PBS, collected and fixed in 10% buffered formaldehyde for 20 minutes, stored in 70% ethanol, washed with PBS and then put in Histogel. Spheroids were then dehydrated in graded alcohols, cleared in xylene, and embedded in paraffin on Leica ASP300 Tissue processor. 5 µm-thick slices were made on a Leica rotary microtome (Histocore Autocut R) and deposited on coated glass slides to achieve histological analysis. Slides were observed with Axio Imager M2 epifluorescence microscope (Zeiss). Live/dead quantification was performed with QuPath software version 0.7.0.

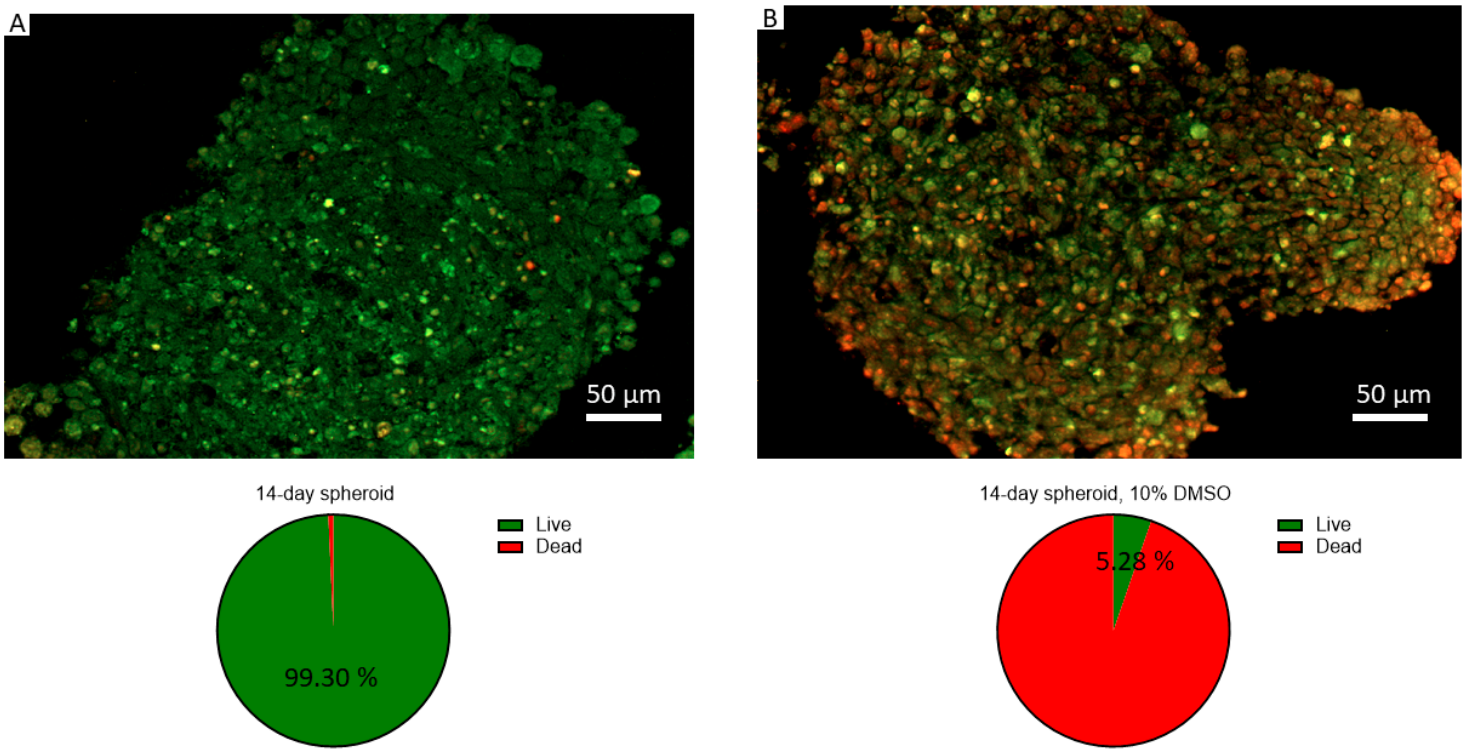


**Supplementary Figure S5**

Viability assay after 14 days of culture. Taste bud spheroids were cultured for 14 days in completed DMEM/F12. Medium was changed with 1% Cyto3D® Live-Dead Assay Kit and spheroids were incubated for 10 minutes (A). Spheroids were incubated with 10% DMSO for 10 minutes as a negative control prior to the assay (B). Live cells are green and dead cells are red. Spheroids were washed 3 x 5 minutes in PBS, collected and fixed in 10% buffered formaldehyde for 20 minutes, stored in 70% ethanol, washed with PBS and then put in Histogel. Spheroids were then dehydrated in graded alcohols, cleared in xylene, and embedded in paraffin on Leica ASP300 Tissue processor. 5 µm-thick slices were made on a Leica rotary microtome (Histocore Autocut R) and deposited on coated glass slides to achieve histological analysis. Slides were observed with Axio Imager M2 epifluorescence microscope (Zeiss). Live/dead quantification was performed with QuPath software version 0.7.0.

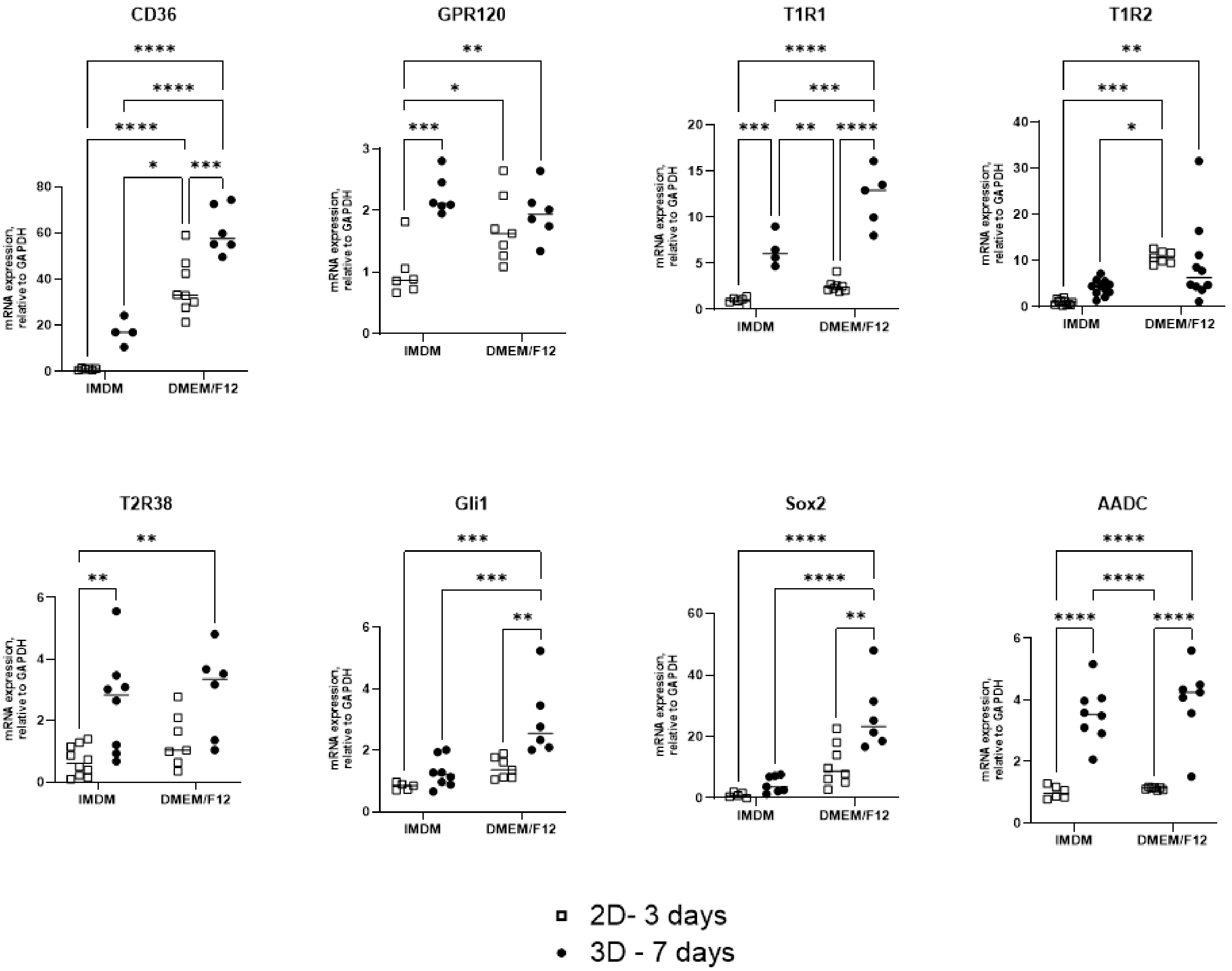


**Supplementary Figure S6**

mRNA expression levels were analyzed by qRT-PCR. Cells were cultured under different conditions: with completed IMDM, in 2D for 3 days or in 3D for 7 days; with completed DMEM/F12, in 2D for 3 days or in 3D for 7 days. Expression of taste receptors CD36, GPR120, T1R1, T1R2, T2R38; of Shh pathway associated genes Gli1 and Sox2; and of serotonin synthesis AADC were normalized to GAPDH and expressed relative to 3 days 2D-cultured cells in IMDM using the 2^-ΔΔCT method. Two-factor ANOVA analysis was performed with Tukey's multiple comparisons test. *p < 0.05, *p < 0.01, ***p < 0.001, ****p<0.0001.
